# Supplementary material for: Immune Cell Networks Uncover Candidate Biomarkers of Melanoma Immunotherapy Response
Source: J Pers Med. 2022 Jun 11;12(6):958. doi: 10.3390/jpm12060958 (PMC9225330; doi:10.3390/jpm12060958)
Supplement: Supplementary file 1 [file jpm-12-00958-s001.zip › SupplementaryMaterial_PROOF.pdf]

Supplementary Information for Vo D, McGleave G & Overton I. 'Immune cell-specific networks uncover candidate markers of cancer immunotherapy response'

Page

Supplementary Figures:

Figure S1. Natural Killer (NK) cell focus network...2

Figure S2. Monocyte focus network..3

Figure S3. Dendritic cell focus network..4

Figure S4. Density plot of pre-filtered RNA-seq data from 49 melanoma patients 5

Figure S5. Survival analysis of BIO_13 in melanoma 6

Figure S6. Kaplan-Meier plots of PDL1 univariate model..9

Supplementary Tables:

Supplementary Tables S1 and S2 are provided separately.

Table S3. Grambsch–Therneau test of the proportional hazards assumption ……………………….. 10

Table S4. Cox proportional hazards model for overall survival in MEL_TCGA training set (n=255) selected by AIC……………………………………………………………………………………………… 10

Supplementary Data (not included in this document):

Supplementary Data File S1. IMMUNET focus networks Cytoscape session.

Supplementary Data File S2. IMMUNET networks.

Supplementary Data File S3. BiNGO results for IMMUNET focus networks.

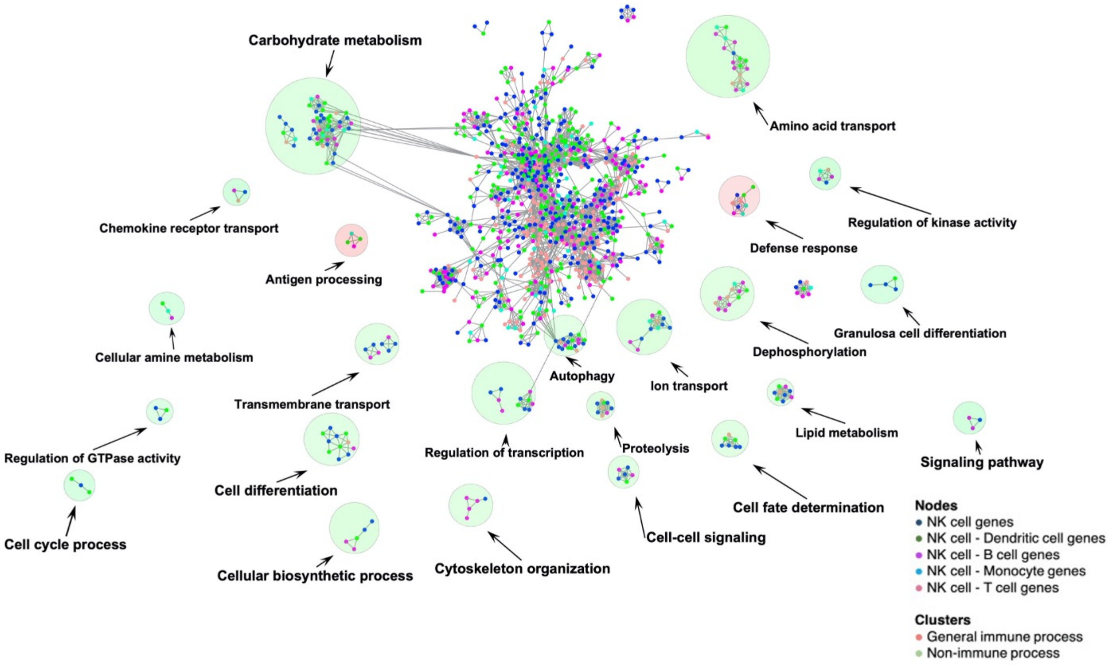

Figure S1. Natural Killer (NK) cell focus network. Results are shown from NetNC analysis of genes found only in NK-net, or in NK-net and one other IMMUNET network. Node colour indicates the representation of each gene in IMMUNET, according to the key. For example, genes shown in blue are only present in NK-net. Clusters are annotated according to significant terms using the BiNGO plugin. Clusters circled in red have immune process annotations, specifically ”Antigen processing” and “Defense response”. Clusters circled in green are annotated with GO terms that are not specific to immune cells.

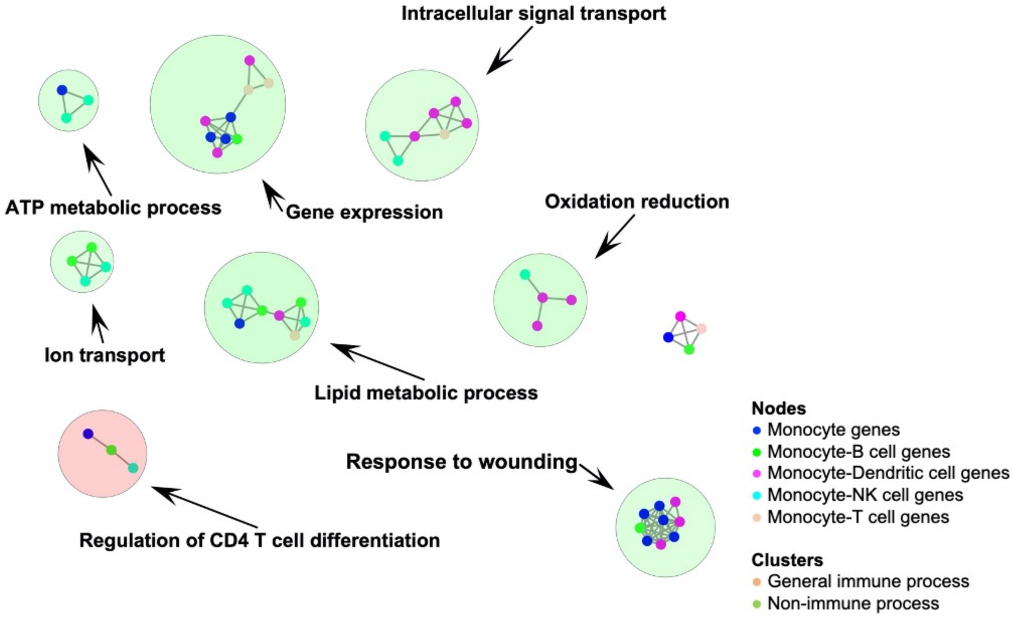

Figure S2. Monocyte focus network. Results are shown from NetNC analysis of genes found only in Mono-net, or in Mono-net and one other IMMUNET network. Node colour indicates the representation of each gene in IMMUNET, according to the key. For example, genes shown in blue are only present in Mono-net. Clusters are annotated according to significant terms using the BiNGO plugin. Clusters circled in red have immune process annotations, specifically “Regulation of CD4 T cell differentiation” and “Response to stress and wounding”. Clusters circled in green are annotated with GO terms that are not specific to immune cells.

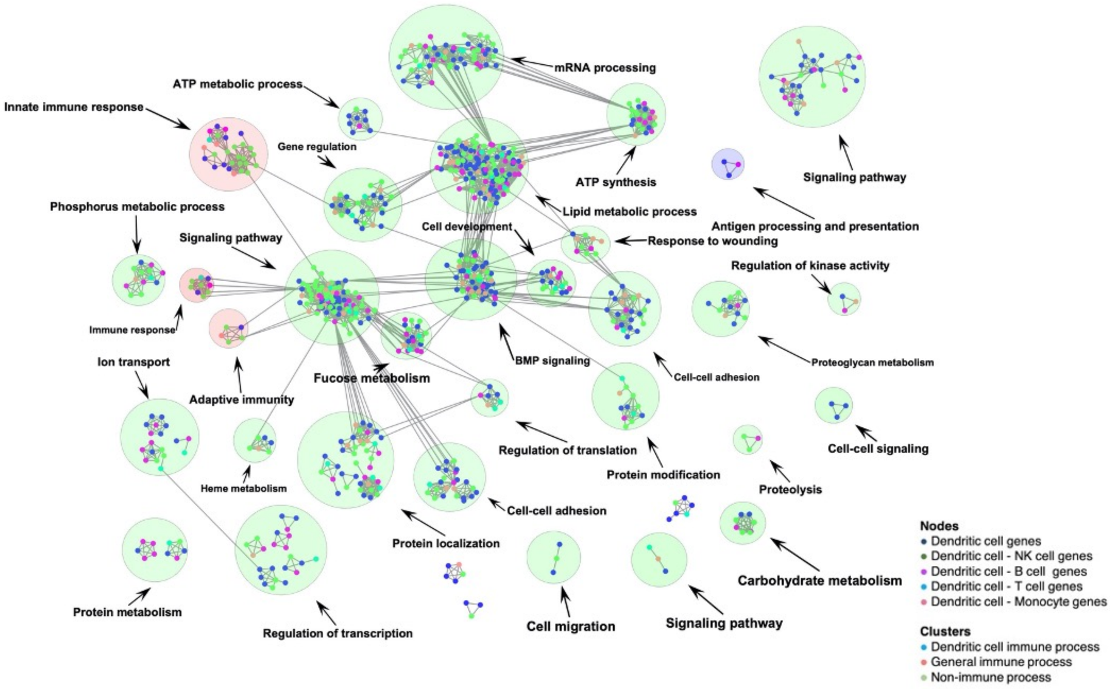

Figure S3. Dendritic cell focus network. Results are shown from NetNC analysis of genes found only in Dend-net, or in Dend-net and one other IMMUNET network. Node colour indicates the representation of each gene in IMMUNET, according to the key. For example, genes shown in blue are only present in Dend-net. Clusters are annotated according to significant terms using the BiNGO plugin and clusters with three more genes are shown. The clusters circled in red have immune process annotations, specifically “antigen processing and presentation”, “adaptive immunity” and “immune response” and a cluster circled in blue . Clusters circled in green are annotated with GO terms that are not specific to immune cells.

A. B.

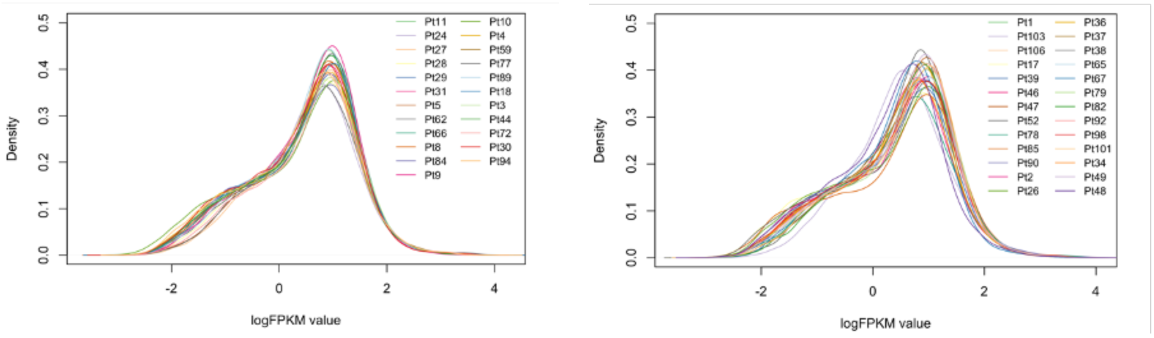

Figure S4. Density plots of pre-filtered RNA-seq data from 49 melanoma patients (Riaz et al. 2017). Gene expression value, FPKM is transformed into log10 value. The density plot of MEL_NAI and MEL_PROG cohorts were shown in (A) and (B), respectively. Each line represents data from one patient which is summarized with the corresponding colour on the right side of each plot.

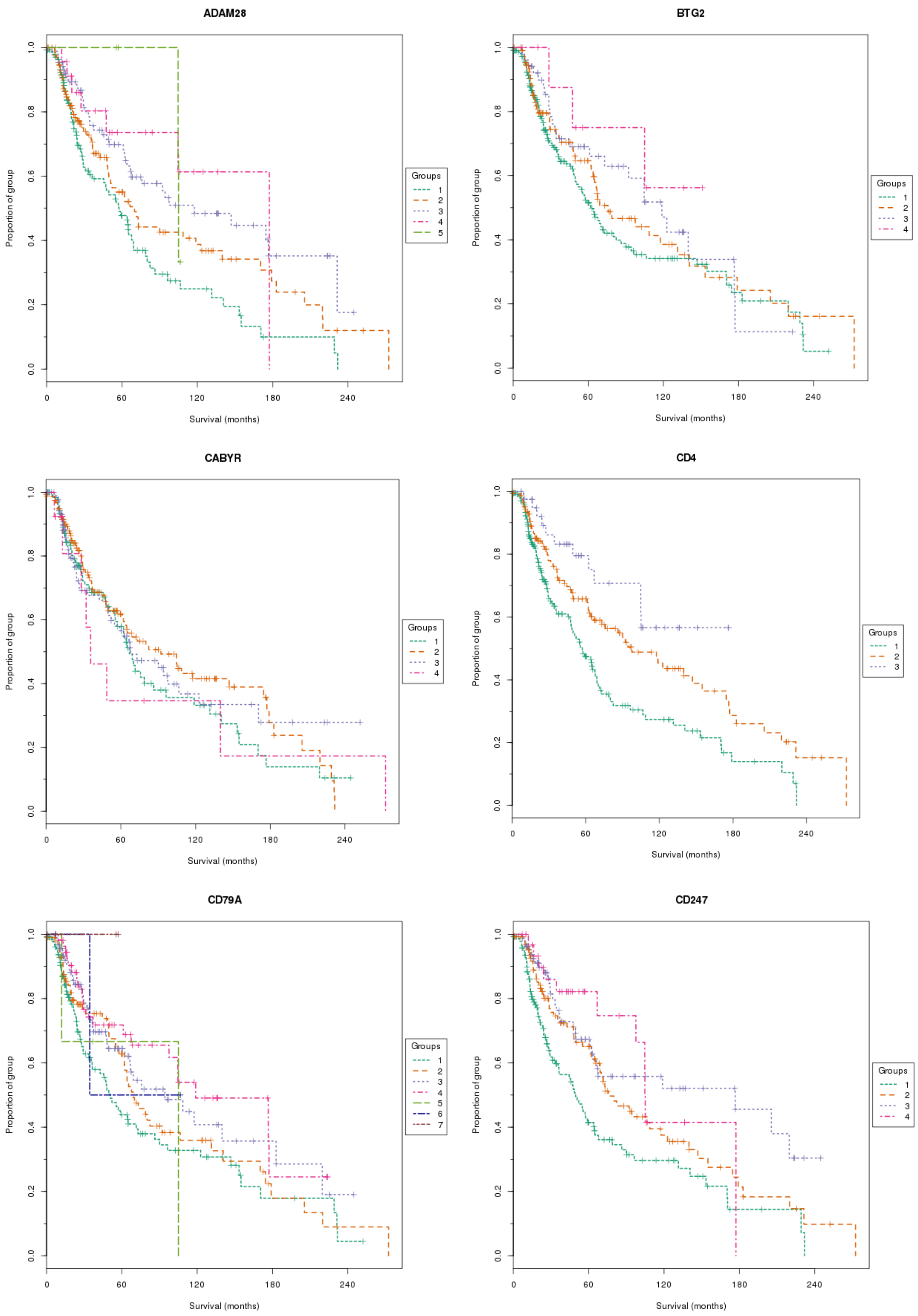

Figure S5. Survival analysis of BIO_13 in melanoma. Continued on the next page.

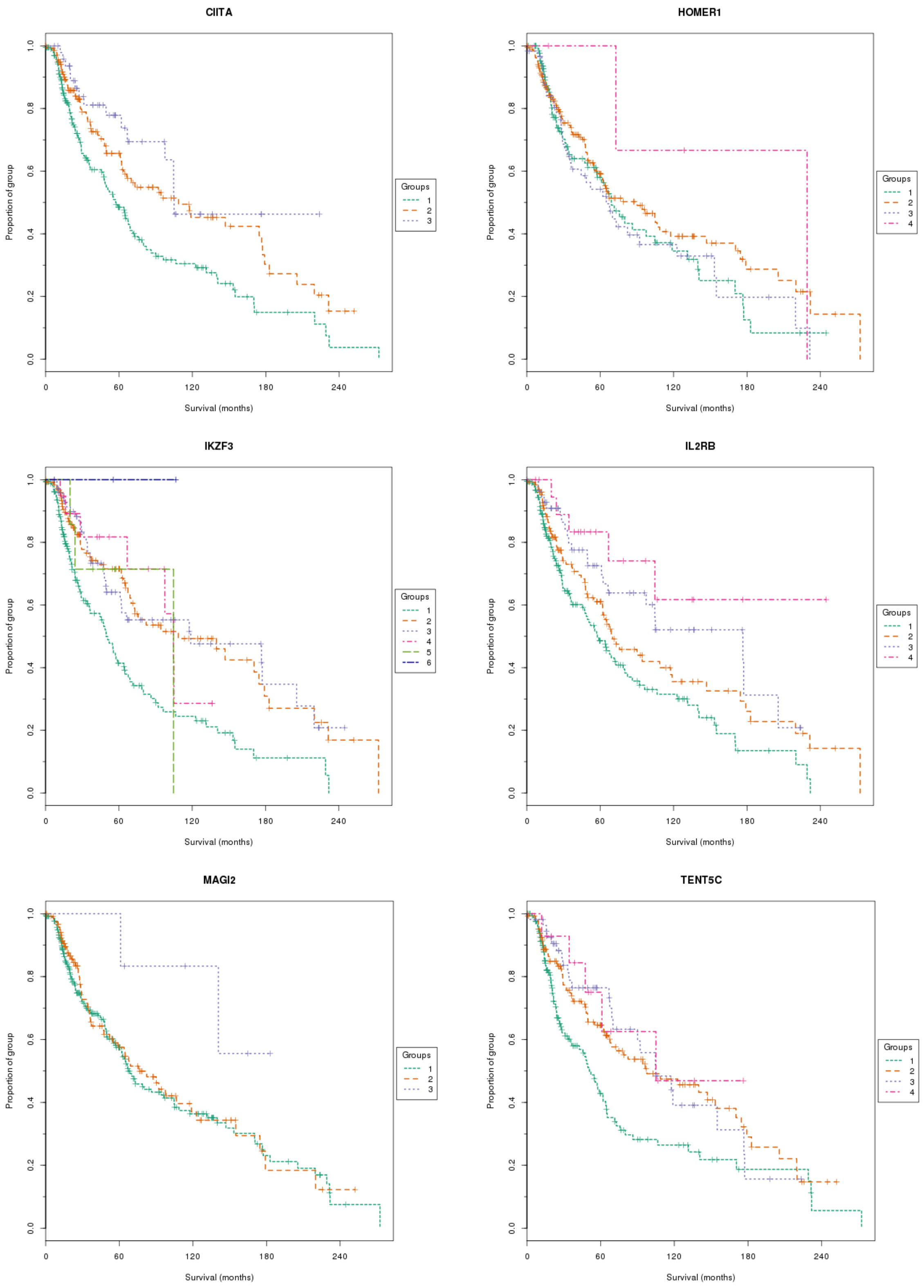

Figure S5. Survival analysis of BIO_13 in melanoma. Continued on the next page.

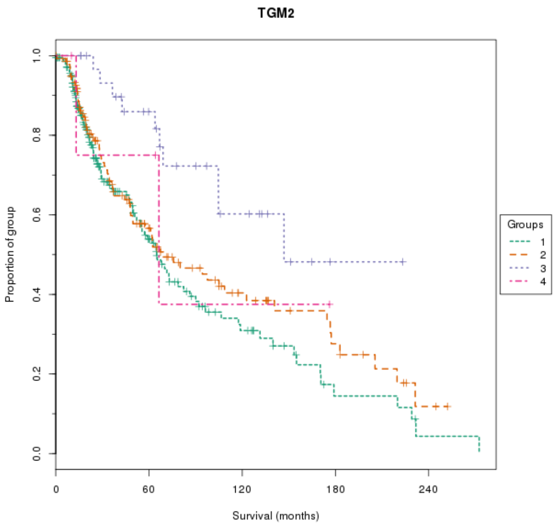

Figure S5. Survival analysis of BIO_13 in melanoma. Groups were defined by Gaussian Mixture Modelling of genes expression values in cutaneous melanoma patients from TCGA (n = 390). Numbering in the key identifies each sub-group; higher numbering corresponds to a higher expression level of the gene analysed. Also see Table 3 in the main manuscript.

A. B.

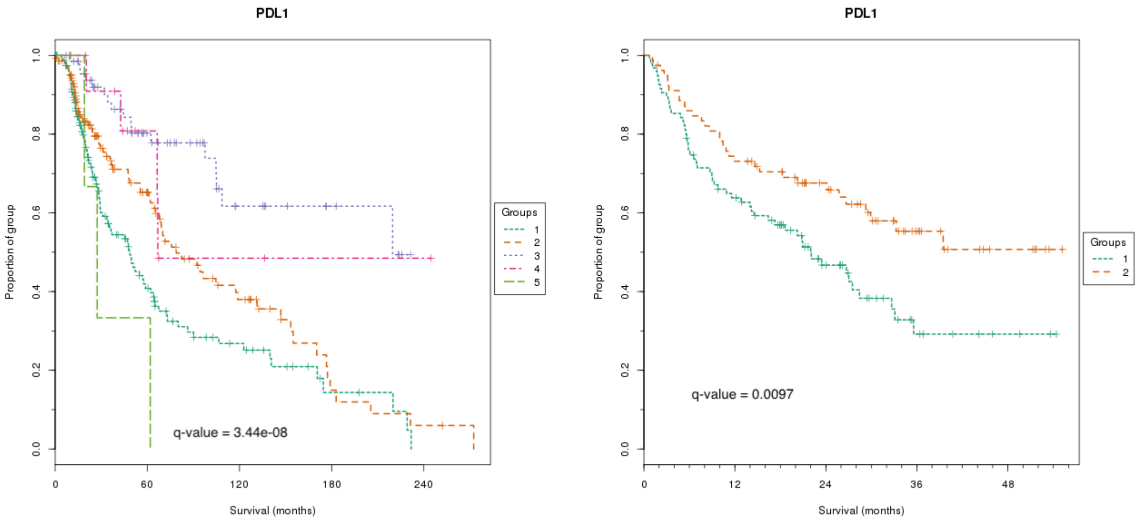

Figure S6. Kaplan-Meier plots of PD-L1 univariate model. A, MEL_TCGA (n=390) cohort. B, MIXED_ICI (n=174). P-value of log-rank test was adjusted by Benjamini-Hochberg correction method. Risk groups were determined by regularised Gaussian mixture modelling.

Prognostic factor	Chi-square test	p-value
Age	2.22E-05	0.996
Tumour stage	3.25	0.072
CIITA	1.22E-03	0.972
IKZF3	1.94	0.163
CD247	1.95	0.163
TENT5C	2.46	0.117
Overall	7.33	0.292

Table S3. Grambsch–Therneau tests of the proportional hazards assumption. Results correspond to the multivariate model shown in Table S4, below.

Prognostic factor	p-value	Hazard ratio	95% Confidence interval
Age	9.64E-03*	1.0176	1.00 – 1.03
Tumour stage	8.66E-03*	1.3603	1.08 – 1.71
CIITA	1.04E-02*	0.8514	0.75 – 0.96
IKZF3	4.23E-02*	1.2369	1.01 – 1.52
CD247	1.21E-01	0.7876	0.58 – 1.07
TENT5C	6.65E-02	0.9377	0.88 – 1.00

Table S4. Cox proportional hazards model for overall survival in MEL_TCGA training set (n=255) selected by AIC. Asterisks (*) indicates p-value < 0.05. Significant variables were taken forwards into the multivariate model shown in Table 4 (main manuscript).
